# Progress in Medicine

**Published:** 1902-10-18

**Authors:** 


### Progress in Medicine.

**Small-pox and Vaccination.**—The study of the epidemiology of small-pox is rendered very difficult by the unique position occupied by the disease, the incidence and the mortality of which have been greatly modified by vaccination. As a result of such a study Newsholme[21] concludes that the chief governing factor is personal infection, that the influence of climatic conditions is not clear, for though commoner in dry years, small-pox does not follow the curve of small rainfall as scarlet and rheumatic fever and diphtheria do. Major epidemics show no regular periodicity, but a fairly regular periodicity, considerably disturbed by vaccination, is shown by minor epidemics, but there must be some further undiscovered factor, some "epidemic constitution," which determines why small-pox spreads more rapidly in some years than in others. E. J. Edwardes[22] shows conclusively by a mass of statistics the value of vaccination, and also of revaccination, as shown especially by the results of revaccination in Germany. He points out that epidemics used to recur about every three or four years, that is, directly the susceptible element of the population—the children born since the last epidemic—acquired any significance. The case for revaccination is strengthened by A. K. Chalmer's[23] account of the recent Glasgow epidemic. The machinery for enforcing vaccination and isolation is reviewed by Mrs. Garrett Anderson.[24] While the administration of all other sanitary matters is vested in borough councils, municipal corporations, or urban and district rural councils, vaccination is dealt with by the Poor-law guardians. J. C. MacVail,[25] in making important suggestions for future legislation, contends that this severance of vaccination from other sanitary matters should be remedied, a general standard of efficient vaccination should be enforced, and all lymph should be supervised, if not actually supplied, by Government. Revaccination should be placed on the same footing as primary vaccination, a measure which would meet the isolation hospital difficulty by largely removing the necessity for such hospitals. T. Colcott Fox[26] treats of the complications of vaccinia. Given pure lymph, asepsis, and care these are practically *nil*, although syphilis, tetanus, and other diseases may be inoculated by accidental contamination, and natural variations in results occur. Inflammatory reaction is apt to be more marked in revaccination and with the use of bovine lymph. The red excrescence known as "raspberry sore" may appear. Cope[27] says it is not protective and ascribes it to feebly acting lymph, and also says in his description of vaccination done with Government lymph—the preparation of which in the establishments of the Local Government Board is described by F. R. Bloxall[28]—that the œdema observed in revaccination depends on affection of the axillary glands, and quickly subsides if these are fomented. Movement of the arm greatly contributes to œdema. W. McWanklyn[29] treats of the differential diagnosis of small-pox and chicken-pox, and lays stress on the presence, in the initial fever of variola, of a general muscular flaccidity and tonelessness leading to a dull, fatigued expression of face. The terms "shotty" and "umbilicated" are misleading and should be discarded, but the more superficial, delicate, and irregular character of the varicella pock is of great importance, as also is the distribution of the rash. In varicella, the forearms and hands and the legs and feet are but slightly, if at all, affected by the rash. This point is of cardinal importance in diagnosis. A case, however, diagnosed after some difficulty as chicken-pox, is reported by W. M. Young[30] in which a free eruption appeared on the backs of the wrists as well as on the face. The patient was taking bromide at the time. Much can be written on the subject of the bacteriology of vaccinia and variola,[31] but the question remains as yet unsolved. H. Roger[32] describes some small roundish bodies which he has discovered in variolous pustules, and which he considers specific. The leucocytosis present in variola he describes as chiefly of a large mononuclear type. A Special Commission[33] appointed by the *Lancet* for that purpose report on the twelve samples of commercial lymph examined by them, including that supplied by the Local Government Board. All were potent, but in all extraneous organisms in varying quantity were found. The lymphs are tabulated according to their freedom from these organisms on which depend the concomitant inflammatory phenomena of vaccinia. "The best results are invariably obtained with those lymphs which produce late vesiculation." "If a lymph be free from bacteria, especially streptococci and staphylococci, and if typical vesicles, slow of development with little inflammatory areolæ and late in coming to maturity, be obtained" the patient is properly vaccinated. Over-glycerinated rather than under-glycerinated lymph is preferable, even at the risk of having to make a second insertion. Glycerination carried to the point of destruction of practically all bacteria present does not necessarily impair the efficiency of the vaccine. It appears to be at least as difficult to obtain bacteria-free lymph in America as in this country,[34] and manufacturers are warned against issuing "green" lymph and practising economy by relying on the destructive power of glycerine and so lessening their antiseptic precautions.[35] Acland and Newsholme[36] show by tables that there is a relative excess of juvenile small-pox in places showing a high rate of vaccination default, and the former[37] urges as a matter of common sense that the nation should decide for sufficient vaccination with good lymph. Mr. A. F. Burridge[38] in a paper read before the Institute of Actuaries, of which he is vice-President, emphasised the important point that "while the mortality in infancy has been reduced to one-fortieth of its former rate, the mortality for ages "45 and upward" has not been diminished. Returns culled from the Registrar General's report[39] for the present epidemic show a mortality of only 0·7 per cent. for vaccinated children under 10 as against 57·3 per cent. for unvaccinated children under 10. J. C. Thresh[40] considers carefully the incidence of small-pox in the Orsett Union of Essex, and especially at Purfleet, nearest to the hospital ships. He reaches the firm conviction that small-pox infection is carried by aerial convection even to a distance of two or three miles. The more concentrated the focus of infection the greater the danger. A similar theory was propounded many years ago by W. H. Power[41] with regard to the Fulham Small-pox Hospital. The hospital ships will probably be soon discontinued, as the Metropolitan Asylums Board hopes soon to have 3,200 beds available for small-pox.[42] A cemetery for burial of the dead has been prepared at the new Joyce Green Hospital. Medical supervision of "contacts" is preferable to isolation, as a rule. A wise measure would be provision for reception of contacts who develop febrile illness within 14 days of coming under observation.[43]

21 Brit. Med. Jour., July 5. 22 Ibid. 23 Ibid 24 Ibid. 25 Ibid. 26 Ibid. 27 Ibid. 28 Ibid. 29 Ibid. 30 Lancet, June 7, p. 1598. 31 Brit. Med. Jour.. July 5. 32 Brit. Med. Jour. Epitome, March 15. 33 Lancet, June 7. 34 Med. Rec., March 18, p. 391. 35 Academy of Med., March 22, p. 563. 36 Brit. Med. Jour., June 14, p. 1510. 37 Ibid., April 26. 38 Ibid., May 3. 39 Ibid., April 26. 40 Lancet, Feb. 22 and April 26. 41 Ibid., Feb. 22, p. 534. 42 Ibid., March 1. 43 Ibid., March 8. p. 678.

*(To be continued.)*

